# Inhibition of STAT6 Activation by AS1517499 Inhibits Expression and Activity of PPARγ in Macrophages to Resolve Acute Inflammation in Mice

**DOI:** 10.3390/biom12030447

**Published:** 2022-03-14

**Authors:** Ye-Ji Lee, Kiyoon Kim, Minsuk Kim, Young-Ho Ahn, Jihee Lee Kang

**Affiliations:** 1 Department of Physiology, College of Medicine, Ewha Womans University, Seoul 07804, Korea; shyzizibe@naver.com (Y.-J.L.); yoonkky@ewha.ac.kr (K.K.); 2 Inflammation-Cancer Microenvironment Research Center, College of Medicine, Ewha Womans University, Seoul 07804, Korea; ms@ewha.ac.kr (M.K.); yahn@ewha.ac.kr (Y.-H.A.); 3 Department of Pharmacology, College of Medicine, Ewha Womans University, Seoul 07804, Korea; 4 Department of Molecular Medicine, College of Medicine, Ewha Womans University, Seoul 07804, Korea

**Keywords:** AS1517499, annexin A1, PPARγ, macrophages, acute peritonitis

## Abstract

Signal transducer and activator of transcription 6 (STAT6) promotes an anti-inflammatory process by inducing the development of M2 macrophages. We investigated whether modulating STAT6 activity in macrophages using AS1517499, the specific STAT6 inhibitor, affects the restoration of homeostasis after an inflammatory insult by regulating PPARγ expression and activity. Administration of AS1517499 suppressed the enhanced STAT6 phosphorylation and nuclear translocation observed in peritoneal macrophages after zymosan injection. In addition, AS1517499 delayed resolution of acute inflammation as evidenced by enhanced secretion of pro-inflammatory cytokines, reduced secretion of anti-inflammatory cytokines in PLF and supernatants from peritoneal macrophages, and exaggerated neutrophil numbers and total protein levels in PLF. We demonstrate temporal increases in annexin A1 (AnxA1) protein and mRNA levels in peritoneal lavage fluid (PLF), peritoneal macrophages, and spleen in a murine model of zymosan-induced acute peritonitis. In vitro priming of mouse bone marrow-derived macrophages (BMDM) and peritoneal macrophages with AnxA1 induced STAT6 activation with enhanced PPARγ expression and activity. Using AS1517499, we demonstrate that inhibition of STAT6 activation delayed recovery of PPARγ expression and activity, as well as impaired efferocytosis. Taken together, these results suggest that activation of the STAT6 signaling pathway mediates PPARγ expression and activation in macrophages to resolve acute inflammation.

## 1. Introduction

Several members of the signal transducer and activator of transcription (STAT) family are involved in regulating macrophage functional status. In particular, STAT6 is the signal mediator of interleukin (IL)-4 and IL-13, promoting an anti-inflammatory process by inducing the development of T helper (Th) 2 lymphocytes and M2 type macrophages [1,2,3,4,5]. Activation of STAT6 is initiated by binding of IL-4 and IL-13 to their receptors, which leads to the activation of Janus tyrosine kinases (JAKs), which are associated with the cytoplasmic tails of the receptors. STAT6 phosphorylation leads to dimerization followed by translocation to the nucleus where STAT6 regulates gene expression [6,7,8]. In addition, macrophage M2 polarization involves tyrosine phosphorylation and activation of STAT6, which mediates the transcriptional activation of M2 macrophage-specific genes such as arginase 1 (Arg1), macrophage mannose receptor (MMR, also known as Mrc1), resistin-like molecule α (Retnla, Fizz1), chitinase-like protein 3 (Chil3, Ym1), and the chemokine genes Ccl17 and Ccl24 [9]. Recent studies reported that anti-inflammatory proteins, including TNF-α-stimulated gene (TSG)-6 and annexin A1 (AnxA1), are also involved in STAT6 signaling in macrophages [10,11], which are also essential for macrophage differentiation. Szanto et al. demonstrated that STAT6 mediates IL-4-induced augmentation of peroxisome proliferator-activated receptor gamma (PPARγ) expression and acts as a facilitating factor for PPAR-mediated transcription in immune cells [12]. Importantly, Liao et al. [1] showed that transfection of STAT6 induced *PPARg* promoter-driven luciferase activity in RAW264.7 cells, suggesting a possibility that STAT6 signaling enhances PPARγ expression via the inductive effect of STAT6 on the *PPARg* promoter. In addition to a role as a major regulator of fatty acid synthesis and storage and glucose metabolism [13], PPARγ and its ligands have been reported to participate in the anti-inflammatory response by mediating M2 programming [14,15].

STAT6 has been reported to regulate systemic inflammation and protect against lethal endotoxemia as *Stat6* deficient mice exhibited enhanced nuclear factor-κB (NF-κB) activation in the liver and lungs [16]. In our previous study, we demonstrated that STAT6 plays a fundamental role in the resolution of acute sterile inflammation since *Stat6* knockout mice displayed enhanced zymosan-induced pro-inflammatory cytokine production along with downregulation of PPARγ signaling [17]. However, how STAT6 phosphorylation is triggered during these pathologic conditions has not been fully established. In particular, the role of STAT6 activation in macrophages remains to be elucidated in inflammatory disease models.

Thus, in the present study, we investigated whether modulating STAT6 activity using a selective STAT6 inhibitor, AS1517499, influences the inflammatory response in zymosan-induced peritonitis by regulating PPARγ expression and activity. We observed temporal increases in AnxA1 protein and mRNA levels in peritoneal lavage fluid (PLF), peritoneal macrophages, and spleen after zymosan injection. In vitro priming of mouse bone marrow-derived macrophages (BMDM) and peritoneal macrophages with AnxA1 induced STAT6 activation and PPARγ expression and activity. We also found that pharmacologic inhibition of STAT6 activity delayed recovery of PPARγ expression and activation in peritoneal macrophages. Inhibition of STAT6 activity also impaired efferocytic ability of macrophages and augmented acute inflammation in vivo.

## 2. Materials and Methods

### 2.1. Reagents

The following reagents were used throughout this study: zymosan (Sigma-Aldrich, St. Louis, MO, USA), AS1517499 (Axon Medchem BV, Groningen, Netherlands), and recombinant mouse AnxA1 (ab202184; Abcam, Cambridge, UK). For ELISA, we used the mouse TSG-6 ELISA kit (RayBiotech Life, Peachtree Corners, GA, USA) and mouse AnxA1 ELSIA kit (ab264613; Abcam, Boston, MA, USA). The antibodies used for Western blotting were as follows: anti-AnxA1, anti-phospho STAT6 (Tyr-641), anti-STAT6, anti-PPARγ, anti-CD36, anti-MMR, and anti-Arg1 from Cell Signaling Technology (Danvers, MA, USA) and anti-β-actin from Sigma-Aldrich. The Pierce BCA protein assay kit was purchased from Thermo Fisher Scientific (Rockford, IL, USA). The gene-specific relative RT-PCR kit was obtained from Invitrogen Life Technologies (Carlsbad, CA, USA). M-MLV reverse transcriptase was obtained from Enzynomics (Seoul, Korea). The Klenow fragment of DNA polymerase and dNTPs were obtained from Intron Biotechnology (Seoul, Korea).

### 2.2. Animal Protocols

Pathogen-free male BALB/c mice (6–8 weeks old weighing 19–21 g) were purchased from Orient Bio (Sungnam, Korea). The Animal Care Committee of the Ewha Medical Research Institute approved the experimental protocol (ESM16-0461). Mice were cared for and handled in accordance with the National Institutes of Health Guide for the Care and Use of Laboratory Animals.

### 2.3. Induction of Acute Sterile Inflammation and Treatment

Mice were administered intraperitoneally with 1 mg zymosan in 500 μL phosphate-buffered saline (PBS) [18,19]. For the pharmacological inhibition experiments, the STAT6 inhibitor AS1517499 [10 mg/kg; dissolved in 20% dimethyl sulfoxide (DMSO) in saline] was administered intraperitoneally 1 h before zymosan injection [20,21,22]. After the initial dose, AS1517499 or its vehicle (20% DMSO) was administered once more 2 days later. All animals were euthanized at 6, 24, or 72 h after zymosan injection.

### 2.4. Isolation of Peritoneal Lavage Cells and Spleen

Peritoneal lavage (PL) was performed using 1.5 mL aliquots of ice-cold Ca^2+^/Mg^2+^-free phosphate-buffered medium (145 mM NaCl, 5 mM KCl, 1.9 mM NaH_2_PO_4_, 9.35 mM Na_2_HPO_4_, and 5.5 mM dextrose, at pH 7.4) for a total of 10 mL per mouse. The number of neutrophils and peritoneal macrophages in PLF was determined according to their unique cell diameter using an electronic Coulter counter fitted with a cell-sizing analyzer (Coulter Model ZBI with a channelizer 256; Beckman Coulter, Indianapolis, IN, USA) [23,24]. In addition, PL cytospins were stained with the Diff-Quik kit (Dade Behring, Newark, DE, USA) to differentiate PL macrophages and neutrophils [25,26,27]. After PL, the spleen was removed, immediately frozen in liquid nitrogen, and stored at −70 °C.

### 2.5. Preparation of Peritoneal Macrophages

Peritoneal macrophages were cultured (5 × 10^5^ per well in 6-well plates) in serum-free X-VIVO medium (04-380Q, Lonza, Walkersville, MD, USA) for 60 min. Nonadherent cells were removed before isolation of total RNA and protein. Approximately 90–95% of the plastic-adherent cells were identified as macrophages based on morphology.

### 2.6. Measurement of Total Protein in Lavage Samples

PLF sample protein concentration was used as an indicator of blood-peritoneal barrier integrity [17,19,28,29,30]. Total protein content in PLF was measured according to the manufacturer’s protocols (BCA protein assay kit, Thermo Fisher Scientific).

### 2.7. Western Blotting

Spleen homogenates and cell lysates (10–100 μg protein/lane) were separated using an 8–10% SDS-PAGE gel and electrophoretically transferred onto nitrocellulose membranes (Whatman GmbH; Dassel, Germany). The membranes were probed with specific antibodies to phospho-STAT6/SAT6, PPARγ, CD36, MMR, Arg1, AnxA1, or β-actin (1:1000 dilution) for 20 h, followed by the addition of a horseradish peroxidase-conjugated secondary antibodies (1:1000) for 30 min. Target proteins were visualized using an enhanced chemiluminescence detection kit (Thermo Fisher Scientific).

### 2.8. Real-Time Quantitative PCR

Total RNA was extracted using TRIzol reagent (RNAiso plus, Takara Bio Inc., Kusatsu, Japan). Total RNA (1 μg) was reverse transcribed using ReverTra Ace^TM^ qPCR RT Master Mix (Toyobo, Japan). For quantitative PCR reactions, 1:5 dilutions of cDNA products were amplified using SYBR green real time PCR master mix reagent (Toyobo) and analyzed by using Real-Time PCR System (Applied Biosystems, Foster City, CA, USA). Samples were incubated for an initial denaturation at 95 °C for 10 min, then 40 PCR cycles were performed using the following conditions: 95 °C for 15 s, 60 °C for 1 h, 95 °C 15 s, 60 °C 1 h, and 95 °C 15 s. Primer sets for PCR-based amplifications were designed using Primer Express software (Thermos Fisher Scientific, Waltham, MA, USA). The following primers were used: (1) *AnxA1*, forward: 5′-TGTATCCTCGGATGTTGCTGCC-3′, reverse: 5′-CCATTCTCCTGTAAGTACGCGG-3′; (2) *TSG6*, forward: 5′-CTTGGCTGACTATGTAGA-3′, reverse: 5′-TTCCTGTGCTAATGATGT-3′; (3) *PPARg*, forward: 5′-GCCCTTTGGTGACTTTATGG-3′, reverse: 5′-CAGCAGGTTGTCTTGGATGT-3′; (4) *CD36*, forward: 5′-TTGTACCTATACTGTGGCTAAATGAGA-3′, reverse: 5′-CTTGTGTTTTGAACATTTCTGCTT-3′; (5) *MMR*, forward: 5′-AGAAAATGCACAAGAGCAAGC-3′, reverse: 5′-GGAACATGTGTTCTGCGTTG-3′; (6) Arg1, forward: 5′-GTGGGGAAAGCCAATGAAG-3′, reverse: 5′-GCTTCCAACTGCCAGACTGT-3′; (7) *HPRT*, forward: 5′-CAGACTGAAGAGCTACTGTAATG-3′, reverse: 5′-CCAGTGTCAATTATATCTTCAAC-3′. mRNA levels were normalized against *HPRT* mRNA [31] and are reported as fold-change in expression over the control group.

### 2.9. Enzyme-Linked Immunosorbent Assay (ELISA)

For serum cytokine quantification, blood was collected from mice via cardiac puncture and the serum was separated by centrifugation at 1600× *g* for 5 min at 4 °C. The abundance of tumor necrosis factor (TNF)-α, IL-6, macrophage inflammatory protein (MIP)-2, IL-10, hepatocyte growth factor (HGF), AnxA1, and TSG6 was quantitated in cell-free PLF, serum, or cultured macrophage supernatants by ELISA kit (R&D Systems, Minneapolis, MN, USA) following the manufacturer’s instructions.

### 2.10. Immunocytochemistry

Immunocytochemistry was performed on samples obtained by PL cytocentrifugation followed by adherence of peritoneal macrophages to tissue culture dishes. The slides were then fixed with 4% paraformaldehyde, permeabilized with 0.1% Triton X-100 (Sigma-Aldrich), and stained with an anti-macrophage-specific marker (Mac3; BD Pharmingen, San Jose, CA, USA), mouse monoclonal anti-phospho-STAT6, or anti-PPARγ antibody overnight at 4 °C. Subsequently, cells were washed with PBS three times and incubated with fluorescent isothiocyanate-conjugated donkey anti-rabbit IgG (Jackson ImmunoResearch, West Grove, PA, USA). The slides were mounted in Vectashield mounting medium with DAPI (Vector Laboratories, Inc., Youngstown, OH, USA). All slides were imaged using a confocal microscope (LSM5 PASCAL; Carl Zeiss, Jena, Germany) equipped with a filter set with excitation at 488 nm and 543 nm. Phospho-STAT6 and PPARγ staining were quantified by creating masks and measuring the mean fluorescence intensity of each stain using laser scanning microscopy image examiner software (Carl Zeiss).

### 2.11. Induction of Apoptosis

Human T lymphocyte Jurkat cells were obtained from the American Type Culture Collection (Rockville, MD, USA). Apoptosis was induced by ultraviolet irradiation at 254 nm for 10 min. The cells were then incubated for 2 h before use. After irradiation, cells were 70% apoptotic by evaluation of nuclear morphology by light microscopy [32,33].

### 2.12. Efferocytosis Assay

PL cells were isolated and cytospun to assess phagocytic indices [32,34]. The phagocytic index (PI) was calculated using the following formula: [(number of apoptotic bodies)/(200 total macrophages)] × 100. For ex vivo phagocytosis assays, Jurkat T cells were fluorescently labeled with PKH67 (green) prior to induction of apoptosis according to the manufacturer’s instructions (PKH67-Fluorescent Cell Linker Kits for General Cell Membrane Labelling; Sigma-Aldrich). In brief, 5 × 10^5^ Jurkat T cells/mL were washed in serum-free culture medium and resuspended in 2 mL PKH67-containing Diluent C (1 × 10^−7^ M) for 4 min at RT. Non-labeled peritoneal macrophages (10^5^ cells/mL) from mice treated with saline, AS1517499, zymosan, or zymosan + AS1517499 were plated onto coverslips in a 24-well plate. Then, PKH67-labeled apoptotic Jurkat cells were co-cultured with peritoneal macrophages at a 5:1 ratio for 90 min at 37 °C in 500 µL X-VIVO media. Coverslips were washed twice with PBS to remove the non-ingested apoptotic cells. The slides were then fixed with 4% paraformaldehyde and permeabilized with 0.1% Triton X-100 (Sigma-Aldrich). The slides were mounted in Vectashield mounting medium with DAPI (Vector Laboratories, Inc., Youngstown, OH, USA). All slides were imaged using a confocal microscope (LSM5 PASCAL). For each condition, more than 200 alveolar macrophages were randomly observed and scored by two independent blinded observers. Each condition was tested in duplicate, and the reader was blinded to the sample identity during analysis. Efferocytosis of peritoneal macrophages was determined using the phagocytic index.

### 2.13. In Vitro Exposure of BMDM and Peritoneal Macrophages to Stimulants

Primary BMDM were isolated from BALB/c mice as previously described [35]. Briefly, BMDM were differentiated using 20% L929 supernatant containing 10% fetal bovine serum (FBS) from murine bone marrow myeloid stem cells. After 7 days of culture, the differentiation of BMDM was confirmed by fluorescence-activated cell sorting (FACS) analysis using anti-CD11b. BMDM (10^6^ cells/mL) were treated with 1, 10, or 100 ng/mL AnxA1. In addition, mouse peritoneal macrophages were treated with 100 ng/mL AnxA1.

### 2.14. Statistical Analysis

Values are expressed as the means ± standard error of the mean (S.E.M.). Two-way analysis of variance (ANOVA) was performed for comparisons of multiple parameters, and Tukey’s post hoc test was applied where appropriate. Student’s t-test was used for comparisons of two sample means. A *p*-value less than 0.05 was considered statistically significant. All data were analyzed using Prism 5 software (GraphPad Software, Inc., San Diego, CA, USA).

## 3. Results

### 3.1. Administration of AS1517499 Inhibited STAT6 Activation in Peritoneal Macrophages and Spleen after Zymosan Injection

**Figure 1:**
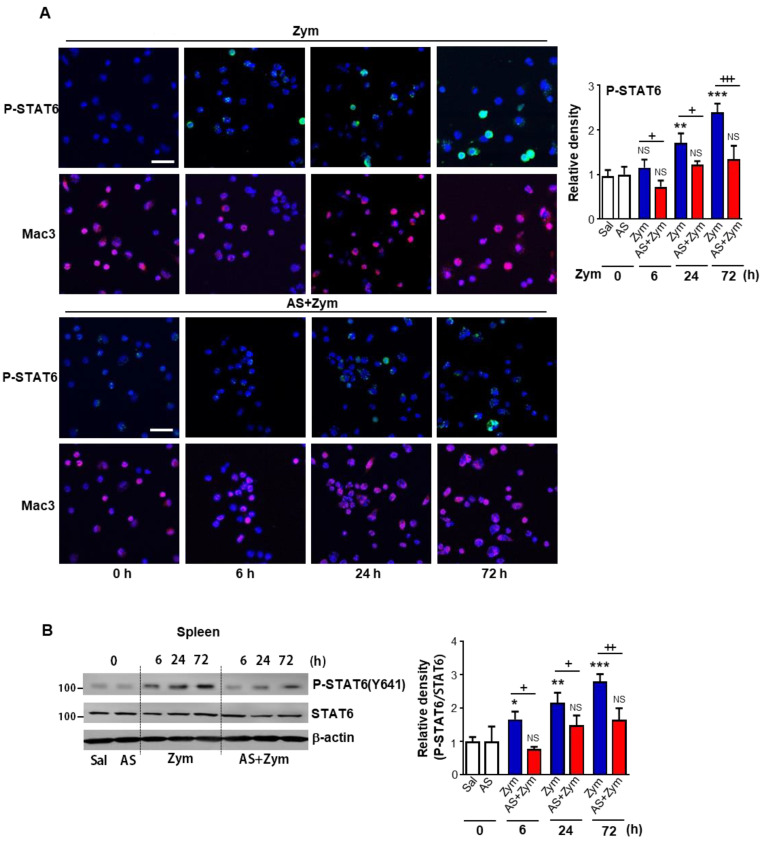
Administration of AS1517499 inhibits STAT6 activation after zymosan injection. Mice were injected i.p. with 1 mg zymosan (Zym) in 500 μL saline. Where indicated, the dose of 10 mg/kg AS1517499 (AS, i.p.) was administered 1 h before Zym injection and once every two days thereafter. (**A**) **Left**: Immunofluorescence staining for phospho-STAT6 (green) and macro-phage-specific marker (Mac3, Red) in peritoneal macrophages (PM) from Zym or AS+Zym-treated mice. Images were captured at 400× magnification. **Right**: Quantification of phospho-STAT6 staining in Mac3-positive macrophages. The imaging medium was Vectashield fluorescence mounting medium containing DAPI. Scale bars = 40 μm. Representative results from three mice per group are shown. (**B**) **Left**: Immunoblots analysis of total and phosphorylated STAT6/total STAT6 in spleen homogenates. **Right**: Densitometric analysis of the relative abundance of phosphorylated STAT6 (pSTAT6) normalized to that of total STAT6. Values represent the means ± SEM of results from five (A) or three mice (B) per group. * *p* < 0.05, ** *p* < 0.01, *** *p* < 0.001 compared with saline control; + *p* < 0.05, ++ *p* < 0.01, +++ *p* < 0.001 for Zym+AS vs. Zym at a given time point (Two-way ANOVA with Tukey’s post hoc test).

Resident peritoneal macrophages play a key role in sensing of peritoneal injury and the regulation of neutrophil infiltration [36]. Injection of zymosan can induce an acute peritonitis and cause microscopic and functional alterations in distant organs [17,19,37,38]. In particular, it was suggested that the spleen is the most pronounced location of phagocytes carrying ingested zymosan [37]. Thus, we first analyzed STAT6 abundance and activation in peritoneal macrophages and spleen after i.p. injection of zymosan. Consistent with findings from our previous study [17], dual immunofluorescence results revealed that STAT6 phosphorylation (green) in Mac3-positive peritoneal macrophages (red) was enhanced after peritoneal injection of zymosan in a time-dependent manner (Figure 1A). Enhanced nuclear localization of phosphorylated STAT6 staining was also observed, indicating active STAT6 signaling. However, administration of the specific STAT6 inhibitor AS1517499 before zymosan injection reversed the enhanced phosphorylation of STAT6 observed in peritoneal macrophages at each time point following zymosan treatment. Furthermore, western blot analysis showed lower phosphorylated STAT6 levels in the spleen at each time point following zymosan injection (Figure 1B). Phosphorylated STAT6 was barely detectable in mice treated with saline or AS1517499 alone in peritoneal macrophages or spleen. These data suggested that AS1517499 effectively inhibited the enhanced STAT6 activation in peritoneal macrophages and spleen observed in mice following zymosan injection.

### 3.2. AS1517499 Enhanced Pro-Inflammatory Cytokine Production and Reduced Pro-Resolving Inflammatory Cytokines 

**Figure 2:**
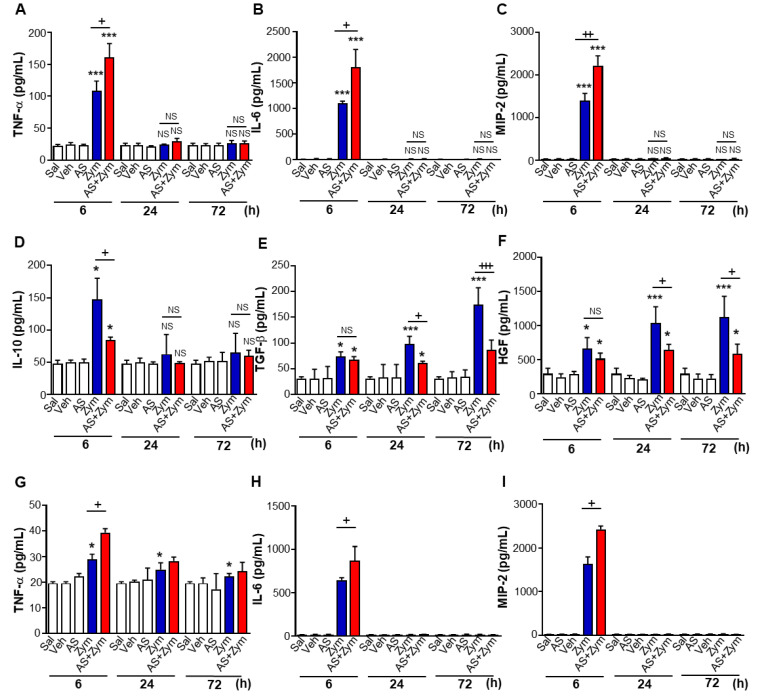

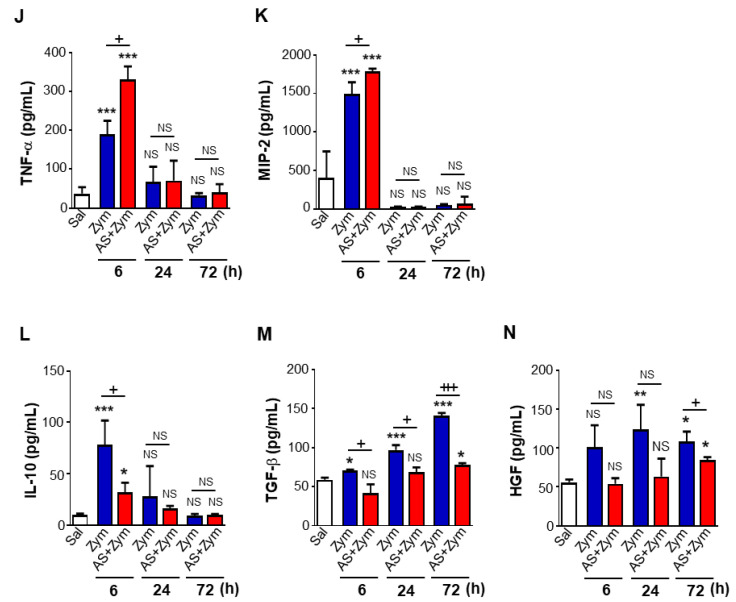
Administration of AS1517499 enhances pro-inflammatory mediators but reduces anti-inflammatory mediators after zymosan treatment. Peritonitis was induced as in Figure 1. Where indicated, the dose of 10 mg/kg AS1517499 (AS, i.p.) was administered 1 h before zymosan (Zym) injection and once every two days thereafter. (**A**–**F**) ELISA of TNF-α, IL-6, MMP-2, IL-10, TGFβ, and HGF protein levels in the peritoneal lavage fluid (PLF). (**G**–**I**) ELISA of TNF-α, IL-6, and MMP-2 in serum. (**J**–**N**) ELISA of TNF-α, MIP-2, IL-10, TGFβ, and HGF in peritoneal macrophages (PM) cultured media. Values represent the means ± SEM of five (**A**–**E**) or three mice (**F**–**N**) per group. * *p* < 0.05, ** *p* < 0.01, *** *p* < 0.001 compared with saline control; + *p* < 0.05, ++ *p* < 0.01, +++ *p* < 0.001 for Zym+AS vs. Zym at a given time point (Two-way ANOVA with Tukey’s post hoc test).

STAT6 has been shown to be a key player in protecting against lethal endotoxemia and zymosan-induced peritonitis [16,18]. To investigate the effect of pharmacologic inhibition of the STAT6 pathway on sterile inflammation, we examined inflammatory responses after zymosan injection with or without AS1517499. Consistent with our previous findings from STAT6^-/-^ mice [17], zymosan-induced increases in TNF-α, IL-6, and MIP-2 levels in PLF were further enhanced by AS1517499 at 6 h after zymosan injection (Figure 2A–C). Notably, AS1517499 reduced the zymosan-mediated increase in IL-10 at 6 h and TGFβ and HGF over the 72-h time period in PLF (Figure 2D–F). Additionally, this enhancing effect of AS1517499 on pro-inflammatory cytokine production in PLF was observed in serum at 6 h after zymosan injection (Figure 2G–I).

We further examined whether pharmacologic inhibition of STAT6 phosphorylation influences pro- and anti-inflammatory cytokine production in cultured peritoneal macrophages. As expected, zymosan-induced TNF-α and MIP-2 levels in the supernatants of peritoneal macrophages were higher in mice treated with zymosan + AS1517499 compared to those in the zymosan only group (Figure 2J,K). However, the levels of IL-10 were lower at 6 h and the levels of TGFβ and HGF were lower up to 72 h in the supernatant of peritoneal macrophages from the zymosan + AS1517499 animals, respectively, compared to those in the zymosan only group (Figure 2L–N).

**Figure 3:**
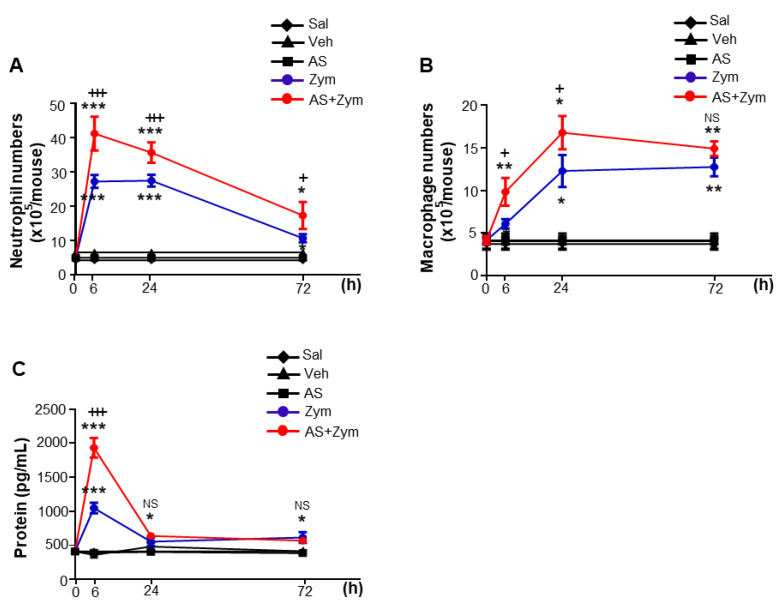
AS1517499 enhances recruitment of inflammatory cells and protein levels over the course of inflammation. Peritonitis was induced as in Figure 1. Where indicated, the dose of 10 mg/kg AS1517499 (AS, i.p.) was administered 1 h before zymosan (Zym) injection and once every two days thereafter. (**A**,**B**) Neutrophil and macrophage numbers in peritoneal lavage fluid (PLF). (**C**) Total protein levels in PLF were analyzed by protein assay kit. Values represent the means ± SEM of five mice per group. * p < 0.05, ** p < 0.01, *** p < 0.001 compared with saline control; *+ p <* 0.05, *+++ p <* 0.001 for Zym+AS vs. Zym at a given time point (Two-way ANOVA with Tukey’s post hoc test).

Similarly, the recruitment of inflammatory cells, such as neutrophils and peritoneal macrophages, into the peritoneal cavity and the total protein in PLF were further enhanced by AS1517499 administration for up to 72 and 24 h (Figure 3A,B) and 6 h after zymosan injection, respectively (Figure 3C). Differential cell counts of neutrophils and alveolar macrophages in cytospun PL taken 6 and 24 h after zymosan treatment were similar for each experimental group (data not shown). However, no differences were observed in the levels of pro- and anti-inflammatory cytokines, inflammatory cell counts, and total protein in the peritoneal fluid or serum in mice treated with AS1517499, vehicle only, or saline only. In particular, the time interval for 50% reduction of the maximal recruitment of neutrophils (the resolution interval R*_i_*) was significantly greater in mice treated with zymosan + AS1517499 (50 ± 1.46 h) than in zymosan only–treated mice (32 ± 1.62 h), suggesting a proresolving role of STAT6 [39].

### 3.3. AnxA1 Was Involved in STAT6 Phosphorylation by Macrophages

**Figure 4:**
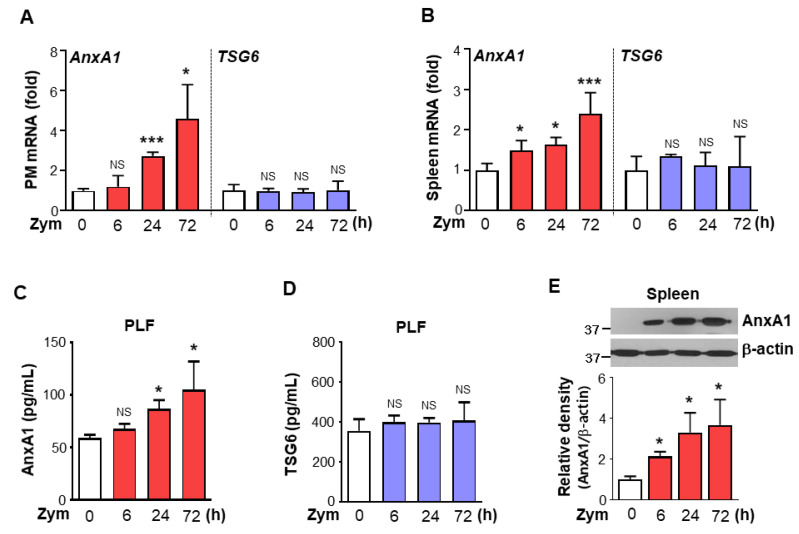
Annexin A1 production is enhanced during zymosan-induced inflammation. Mice were injected i.p. with zymosan (Zym) and peritoneal lavage supernatant, peritoneal macrophages (PM), and spleens were collected at 6, 24, or 72 h after Zym injection. (**A**,**B**) The levels of Annexin A1 (AnxA1) and TNF-α-stimulated gene-6 (TSG6) mRNA over time in PM and spleens analyzed by real-time PCR and normalized to that of hypoxanthine guanine phosphoribosyl transferase (Hprt) mRNA. (**C**,**D**) The abundance of AnxA1 and TSG6 in peritoneal lavage fluid (PLF) as assessed by ELISA. (**E**) Immunoblots analysis of AnxA1 in spleen homogenates. Lower pannel: Densitometric analysis of AnxA1 normalized to that of β-actin. Values represent the means ± SEM of results from five (**A**,**B**) or three mice (**C**–**E**). * *p* < 0.05, *** *p* < 0.001 compared with control at a given time point (Student’s *t*-test).

Data from our previous study suggest that IL-4 and IL-13 are not required for STAT6 activation following zymosan injection [17]; treatment with this glucan did not affect the mRNA and protein levels of these cytokines in peritoneal macrophages and spleen in PLF. Recent data from other laboratories indicate that TSG6 and AnxA1 may be involved in STAT6 signaling and active efferocytosis by macrophages [10,11]. Thus, in the present study, we examined TSG6 and AnxA1 mRNA and protein levels following zymosan injection. Time-dependent increases in AnxA1 mRNA were observed in peritoneal macrophages and spleen from zymosan-treated mice, whereas Tsg6 mRNA in peritoneal macrophages and spleen remained at a basal level throughout the time course (Figure 4A,B). The protein levels of AnxA1 in PLF from zymosan-treated mice increased in a time-dependent manner up to 3 days after zymosan treatment, whereas TSG6 protein did not change relative to control mice treated with saline (Figure 4C,D). In addition, we found that AnxA1 protein was increased similarly in the spleen (Figure 4E). These data suggest that STAT6 activation occurs in an AnxA1-dependent, TSG6-independent manner following zymosan injection. 

**Figure 5:**
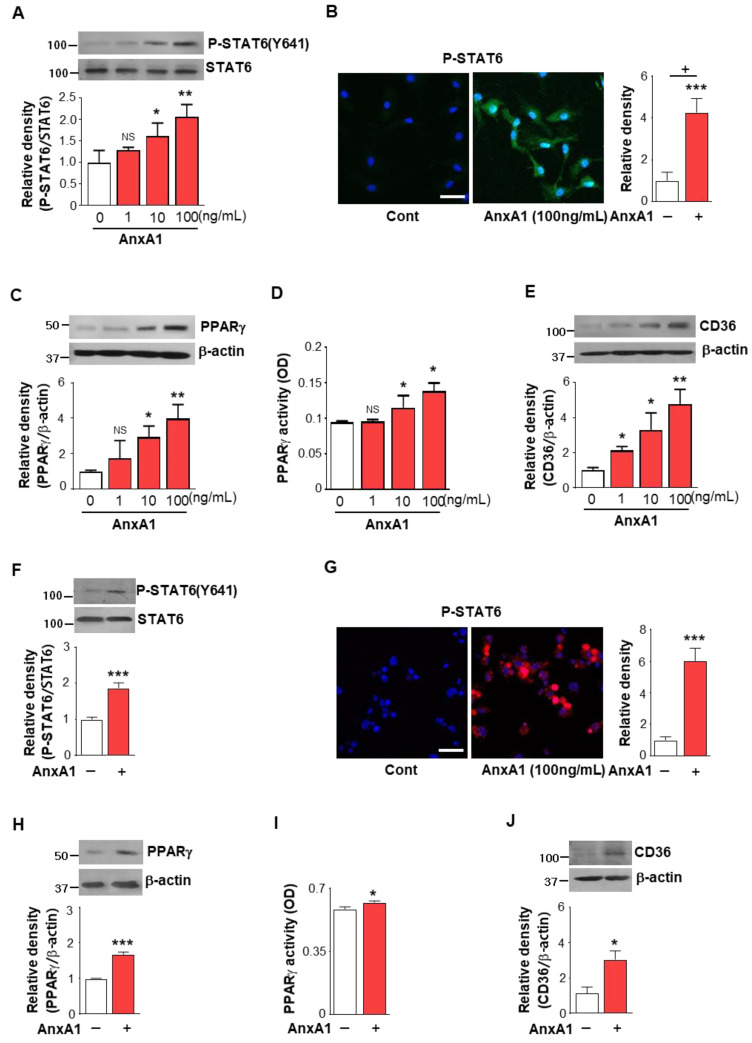
Annexin A1 enhances STAT6 activation and PPARγ expression and activation in BMDM and peritoneal macrophages. Immunoblots analysis of the relative amounts of phosphorylated STAT6/total STAT6 in mouse BMDM (**A**) and peritoneal macrophages (**F**) stimulated with Annexin A1 (AnxA1) for 2 h. Densitometric analysis of the relative abundance of the indicated proteins normalized to that of STAT6. **Left**: Immunofluorescence staining of phosphorylated STAT6 (green) in BMDM (**B**) and peritoneal macrophages (**G**) after 100 ng/mL AnxA1 treatment. The imaging medium was Vectashield fluorescence mounting medium containing DAPI. Original magnification: ×400. Scale bars = 40 μm. Representative results from three independent experiments are shown. **Right**: The ratio of phospho-STAT6 (+) over DAPI (+) macrophages is presented in the bar graph. Immunoblots analysis of the relative amounts of PPARγ, CD36, and β-actin in BMDM (**C**,**E**) and peritoneal macrophages (**H**,**J**) stimulated with AnxA1 for 24 h. Densitometric analysis of the relative abundance of the indicated proteins normalized to that of β-actin. PPARγ activity in nuclear extracts from BMDM (**D**) and peritoneal macrophages (**I**) was analyzed at 24 h after AnxA1 treatment as described in the Methods. Values represent the mean ± SEM of three independent experiments. * p < 0.05, ** p < 0.01, *** p < 0.001 compared with control (Student’s *t*-test).

To assess the direct effect of AnxA1 on the activation of STAT6 and PPARγ in macrophages, we investigated whether AnxA1 enhances the activation of STAT6 and PPARγ in mouse BMDM in vitro. Western blot analysis showed that treatment with mouse recombinant AnxA1 (rAnxA1, 1–100 ng/mL) enhanced STAT6 phosphorylation in BMDM in a dose-dependent manner (Figure 5A). Using confocal microscopy, we confirmed the enhanced nuclear localization of phosphorylated STAT6 following treatment with 100 ng/mL rAnxA1 (Figure 5B). Furthermore, rAnxA1 treatment induced PPARγ expression and activity, as well as enhanced CD36 protein expression (Figure 5C–E). In addition, the enhanced activation of STAT6 and PPARγ were also observed in mouse peritoneal macrophages after 100 ng/mL rAnxA1 treatment (Figure 5F–J). Thus, these in vivo and in vitro data suggest that AnxA1 may be involved in STAT6 activation over the course of acute inflammation following zymosan injection.

### 3.4. AS1517499 Repressed PPARγ Expression and Activation

**Figure 6:**
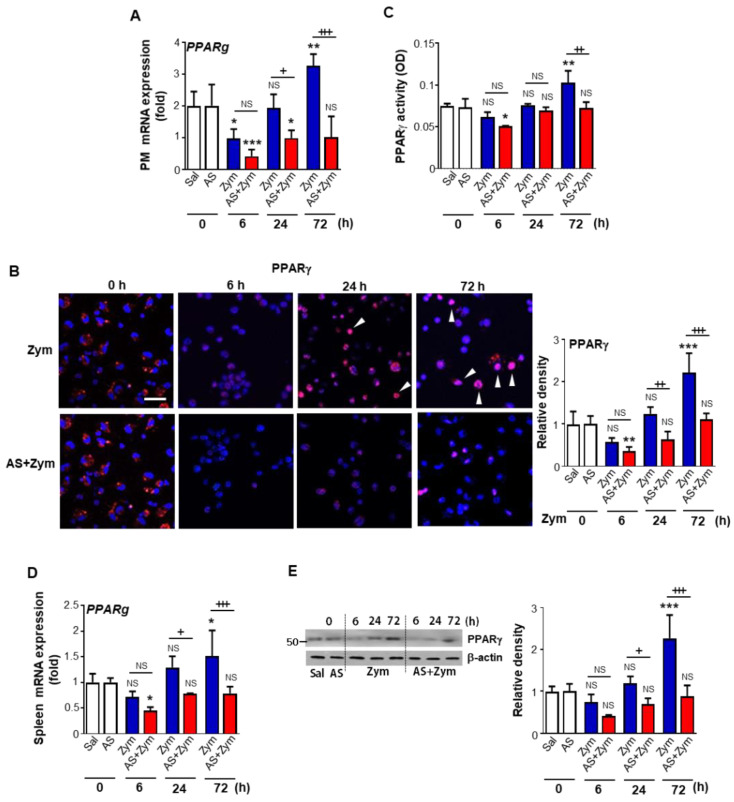
Administration of AS1517499 inhibits PPARγ expression and activation. Peritonitis was induced as in Figure 1. Where indicated, the dose of 10 mg/kg AS1517499 (AS, i.p.) was administered 1 h before zymosan (Zym) injection and once every two days thereafter. (**A**) Changes in the abundance of PPARg mRNAs over time in peritoneal macrophages (PM) analyzed by real-time PCR and normalized to that of hypoxanthine guanine phosphoribosyl transferase (Hprt) mRNA. (**B**) **Left**: Immunofluorescence staining for PPARγ (red) and DAPI (blue) for the nuclei in peritoneal macrophages (PM) from mice treated with zymosan at the indicated times (400× magnification). The imaging medium was Vectashield fluorescence mounting medium containing DAPI. Scale bars = 40 μm. Results are representative of five mice at each time point after zymosan treatment. Right: Quantification of PPARγ staining. (**C**) PPARγ activity in nuclear extracts from PM was analyzed as described in Methods. (**D**) Changes in the abundance of PPARg mRNA over time in spleens analyzed by real-time PCR and normalized to that of Hprt mRNA. (**E**) **Left**: Immunoblots analysis of the abundance of PPARγ protein in spleen samples at the indicated times. β-actin was used as a loading control. **Right**: Densitometric analysis of the relative abundance of PPARγ in each sample. Values represent the mean ± SEM of five (**A**,**B**,**D**) or three mice (**C**,**E**) per group. * p < 0.05, ** p < 0.01, *** p < 0.001 compared with saline control; *+ p* < 0.05, *+*+ *p* < 0.01, *+++ p* < 0.001 for Zym+AS vs. Zym at a given time point (Two-way ANOVA with Tukey’s post hoc test).

Previously, our laboratory and others demonstrated that levels of PPARγ protein expression and downstream M2 markers in peritoneal macrophages are initially reduced at 6 h after zymosan injection before increasing up to 72 h post-treatment [17,18]. In the present study, we examined whether inhibition of STAT6 phosphorylation by AS1517499, a STAT6-specific pharmacological inhibitor, affects PPARγ expression and activation during zymosan-induced peritonitis. Our data showed that PPARg mRNA levels in peritoneal macrophages were decreased by AS1517499 at 24 and 72 h after zymosan treatment relative to the zymosan alone group (Figure 6A). Immunofluorescence staining and confocal microscopy revealed a reduction of nuclear PPARγ staining in peritoneal macrophages in animals treated with zymosan and AS1517499 compared with the zymosan group (Figure 6B). A delayed recovery was observed over the course of inflammation. In parallel, nuclear PPARγ activity in peritoneal macrophages was decreased by AS1517499 after zymosan treatment (Figure 6C). In addition, administration of AS1517499 repressed PPARγ mRNA and protein expression in the spleen (Figure 6D,E) at 24 and 72 h after zymosan treatment relative to the zymosan only group.

**Figure 7:**
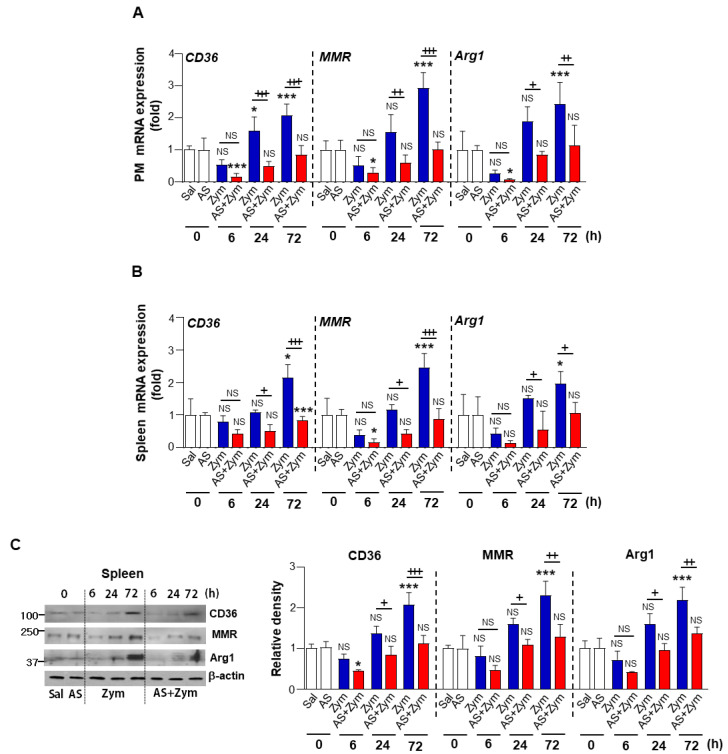
Administration of AS1517499 reduces PPARγ target molecule expression. Peritonitis was induced as in Figure 1. Where indicated, the dose of 10 mg/kg AS1517499 (AS, i.p.) was administered 1 h before zymosan (Zym) injection and once every two days thereafter. (**A**,**B**) Changes in the abundance of *CD36, MMR*, and *Arg1* mRNAs over time in peritoneal macrophages (PM) and spleens analyzed by real-time PCR and normalized to that of hypoxanthine guanine phosphoribosyl transferase (*Hprt*) mRNA. (**C**) **Left**: Immunoblots analysis of the abundance of CD36, MMR, and Arg1 proteins in spleen samples at the indicated times. β-actin was used as a loading control. **Right**: Densitometric analysis of the relative abundance of the indicated protein in each sample. Values represent the mean ± SEM of five (**A**,**B**) or three mice (**C**) per group. * p < 0.05, *** p < 0.001, compared with saline control; *+ p <* 0.05, *++ p <* 0.01, *+++ p <* 0.001 for Zym+AS vs. Zym at a given time point (Two-way ANOVA with Tukey’s post hoc test).

To confirm the changes in PPARγ functional activity, we examined changes in the mRNA and protein levels of CD36, MMR, and Arg1, which are well-established direct transcriptional targets of PPARγ. Similarly, AS1517499 further repressed the decreased expression of these target mRNAs in peritoneal macrophages at 24, and 72 h after zymosan injection. Furthermore, AS1517499 treatment delayed the recovery period of zymosan-induced inflammation (Figure 7A). Spleens from mice treated with zymosan + AS1517499 displayed significantly lower CD36, MMR, and Arg1 mRNA and protein levels compared to mice treated with only zymosan (Figure 7B,C).

### 3.5. AS1517499 Inhibited Efferocytic Ability of Macrophages after Zymosan Injection In Vivo and Ex Vivo

**Figure 8:**
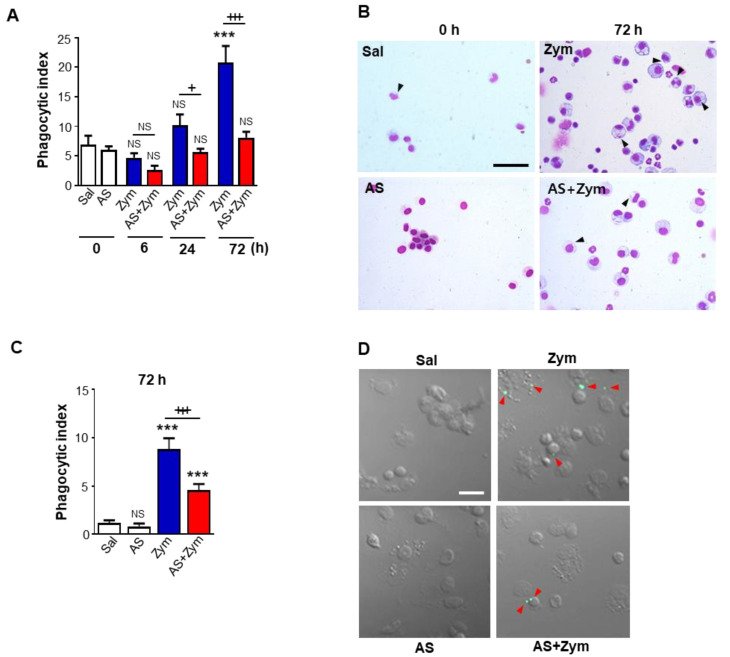
AS1517499 inhibits efferocytic activity of peritoneal macrophages in zymosan-induced peritonitis in vivo and ex vivo. Peritonitis was induced as in Figure 1. Where indicated, the dose of 10 mg/kg AS1517499 (AS, i.p.) was administered 1 h before zymosan (Zym) injection and once every two days thereafter. (**A**,**B**) Peritoneal lavage (PL) was performed, cytospins were stained, and peritoneal macrophage ingestions of apoptotic cells were quantified by calculating a phagocytic index (PI). (**B**) Photomicrographs show macrophage ingestions of apoptotic cells were quantified by calculating a phagocytic index (PI). (**B**) Photomicrographs show cytospin-stained PL cells at 72 h after Zym treatment. (**C**,**D**) Peritoneal macrophages (10^5^ cells/mL) were cultured ex vivo with apoptotic Jurkat cells (5 × 10^5^/mL) labeled with PKH67 (green) for 90 min, and phagocytosis was quantified by calculating a PI. (**D**) Green color represents apoptotic cells that are engulfed by peritoneal macrophages. (**B**,**D**) Original magnification: ×200. Scale bars = 20 μm. Arrowheads indicate peritoneal macrophages with engulfed apoptotic cells or fragments. Values represent the means ± SEM three mice per group. *** p < 0.05 compared with saline control; *+ p <* 0.05, *+++ p <* 0.001 for Zym+AS vs. Zym at a given time point (Two-way ANOVA with Tukey’s post hoc test).

We further evaluated the effects of pharmacologic inhibition of STAT6 phosphorylation on the efferocytic ability of macrophages during zymosan-induced acute peritonitis. To this end, we examined macrophages lavaged from peritonea by microscopy to assess the efferocytosis of endogenous apoptotic cells in vivo. Consistent with our and others’ previous reports [17,18], the phagocytic index (PI) in cytospun peritoneal macrophages progressively increased up to 72 h after zymosan injection (Figure 8A,B). Administration of AS1517499 suppressed the efferocytic ability of endogenous apoptotic cells by peritoneal macrophages following zymosan injection at 24 and 72 h after zymosan treatment compared to mice treated with zymosan only. To confirm the AS1517499-induced defect in efferocytosis by peritoneal macrophages after zymosan injection, freshly isolated peritoneal macrophages from saline-, AS1517499-, zymosan-, or zymosan + AS1517499-treated mice were co-cultured with apoptotic human Jurkat T cells labeled with PKH67 (green) to distinguish them from endogenous apoptotic cells. The PI of macrophages taken from zymosan-treated mice was significantly enhanced ex vivo at 3 days after zymosan treatment compared to saline controls (Figure 8C,D). Uptake by resident peritoneal macrophages in the zymosan + AS1517499 group was significantly decreased relative to those from zymosan only-treated mice. These data suggested that the phagocytic efficiency of peritoneal macrophages significantly increased over the course of inflammation. These results also indicated that phagocytic efficiency may have a relationship with increased efferocytic surface receptors, such as CD36 and MMR, via enhanced activation of the STAT6/PPARγ pathway during zymosan-induced inflammation.

## 4. Discussion

In this study, we examined the role of STAT6 activation in PPARγ activation, peritoneal macrophage efferocytosis, and pro- or anti-inflammatory cytokine production during acute sterile inflammation. To this end, we used the STAT6-specific inhibitor AS1517499 to investigate the effect of pharmacologic inhibition of STAT6 signaling. This inhibitor has been used previously in mouse or rat models of antigen-induced bronchial hyper-reactivity [20], endometriosis [40] neuroinflammation [41], lung fibrosis [21], and carcinoma [42]. Here, we demonstrated that administration of AS1517499 reversed the enhanced STAT6 phosphorylation and nuclear translocation in peritoneal macrophages induced by zymosan injection. This inhibitory effect of AS1517499 on STAT6 phosphorylation was also found in the spleen. These data suggested that AS1517499 administration specifically inhibited the STAT6 pathway in peritoneal macrophages. Similar to our previous study using STAT6-deficient mice [17], here we found that mice treated with zymosan and AS1517499 exhibited higher levels of pro-inflammatory cytokines, including TNF-α, IL-6, and MIP-2, in PLF and serum at 6 h after zymosan injection compared to animals treated with zymosan only. In addition, secretion of pro-inflammatory cytokines, including TNF-α and MIP-2, was enhanced in cultured peritoneal macrophages from AS1517499-treated mice at 6 h after zymosan injection. However, the levels of pro-resolving mediators, such as IL-10, TGFβ and HGF, in PLF and supernatants from peritoneal macrophages were lower in mice treated with zymosan and AS1517499 than in zymosan only-treated mice. Enhanced neutrophil and peritoneal macrophage numbers over the course of inflammation and increased total protein level in PLF at 6 h after zymosan treatment were observed in AS1517499-treated mice compared to control animals. Acute inflammation is most often associated with a neutrophil-rich cellular infiltrate and is generally resolved in a period of days. Notably, the enhanced effect on neutrophil number prolonged until 72 h after zymosan treatment. In particular, the time interval for 50% reduction of the maximal recruitment of neutrophils was greater in the zymosan + AS1517499 group than in zymosan only–treated and control group, suggesting a proresolving role of STAT6 [39]. Taken together, these data support the hypothesis that downregulating STAT6 phosphorylation leads to M1 polarization of macrophages.

Previous studies suggest that IL-4 and IL-13, two cytokines known to induce STAT6 activation, are not involved in STAT6 phosphorylation during zymosan-induced peritonitis [17]. In the present study, we provide novel findings that indicate the possible involvement of AnxA1 in enhanced STAT6 activation over the course of acute inflammation following zymosan injection. Our data demonstrate that AnxA1 mRNA and protein levels in peritoneal macrophages, PLF, and spleen gradually increased over time with a peak response at 72 h after zymosan injection. Importantly, direct positive effects of rAnxA1 on STAT6 activation, as well as PPARγ and CD36 protein levels, were elucidated using an in vitro BMDM and peritoneal macrophage models. Consistent with our findings, published reports have shown that down-modulating AnxA1 expression in BV2 microglial cells resulted in lower levels of STAT6 phosphorylation and impaired phagocytosis of apoptotic cells due to blockade of PPARγ activation [11]. These experiments further suggested that AnxA1-mediated phosphorylation of STAT6 is probably associated with intracellular pathways involving PPARγ and CD36 activities which drive efferocytosis. In addition, AnxA1 has been shown to down-regulate the production of pro-inflammatory mediators (e.g., eicosanoids, nitric oxide, and IL-6), reduce neutrophil migration to inflammatory sites, and promote the clearance of apoptotic granulocytes [43,44]. Nonetheless, further study is necessary to better understand the in vivo mechanism connecting AnxA1 to the STAT6 pathway in macrophages and its role in STAT6 pathway-mediated resolution of inflammation in various inflammatory disease models.

On the other hand, Nepal et al. [10] demonstrated that macrophages secrete TSG6, which is essential for macrophage phenotype transition and promoting the resolution of sepsis-induced acute lung injury [45]. This protein has been demonstrated to mediate efferocytosis via enhanced Gas6 production [10]. Thus, it is possible that enhanced TSG6 could play a critical role in inducing STAT6 phosphorylation in peritoneal macrophages after zymosan injection. However, in the present study, we found that, similar to IL-4 and IL-13 [17], the TSG6 mRNA and/or protein levels remained the same in PLF, peritoneal macrophages, and spleen after zymosan injection, indicating that TSG6 is not involved in the STAT6 pathway during zymosan-induced peritonitis.

Data from several in vitro studies using BMDM from *Stat6* knockout mice and human macrophages demonstrated a role for STAT6 as a facilitator of PPARγ-mediated transcription [12]. More recently, Daniel et al. conducted a genome-wide analysis and determined that IL-4-activated STAT6 drives the extension of the retinoid X receptor cistrome in a PPARγ-dependent manner, providing complex ligand responsiveness in macrophages [46]. Importantly, data from Liao et al. showed that STAT6 signaling enhances *PPARg* expression by inducing the *PPARg* promoter [1]. Our previous study using *Stat6* deficient mice showed that expression of PPARγ and its target genes were decreased at the mRNA and protein levels in peritoneal macrophages and spleen after zymosan injection [17]. Similar to results from this genetic approach, inhibition of STAT6 phosphorylation by AS1517499 reduced the mRNA and protein levels of PPARγ and its activity as assessed by both direct and downstream measures in peritoneal macrophages. The reduced mRNA and protein levels of PPARγ and its target genes by AS1517499 were also observed in spleen at each time point after zymosan injection compared with those in mice treated with zymosan only. Thus, our present data strongly support the in vivo involvement of the STAT6-PPARγ interaction in acute sterile inflammation, as well as the requirement of STAT6 phosphorylation in the recovery of PPARγ expression and activity over the course of zymosan-induced inflammation.

PPARγ transactivation is known to be critical for macrophage efferocytosis and phagocytosis by upregulating the expression of phagocytic receptors and promoting digestion of phagosome and Rac1 activation, including CD36, MMR, Arg1, ATP-binding cassette transporter 1 [47,48,49,50,51], and other recognition receptors [52]. Fernandez-Boyanapalli et al. [18]. demonstrated that efferocytosis by macrophages reflects PPARγ activation during zymosan-induced peritonitis in chronic granulomatous disease. Cai et al. [47] demonstrated in a mouse model of ischemic stroke that *Stat6* deficiency results in a shift of microglia/macrophages toward an adverse phenotype, along with impaired clearance of dead/dying neurons and augmented cerebral inflammation and neuronal death. Similar to *Stat6*-deficient macrophages [17], the efferocytic ability of peritoneal macrophages from AS1517499-treated mice was markedly reduced compared with zymosan only-treated mice, at each time point after zymosan injection. Furthermore, we demonstrated that ex vivo apoptotic cell clearance by peritoneal macrophages taken from AS1517499-treated mice was markedly impaired at 72 h after zymosan injection. Thus, these data suggest that the efferocytic ability of macrophages during acute sterile inflammation could be regulated by modulating the STAT6/ PPARγ pathway.

Accumulating evidence suggests that the process of efferocytosis may resolve inflammation by efficiently clearing dying neutrophils, thus avoiding cellular disruption and the release of inflammatory contents, and by inducing the production of anti-inflammatory mediators such as IL-10, TGF-β, and HGF that dampen pro-inflammatory responses [18,47,53,54]. We previously demonstrated that a PPARγ antagonist (GW9662) can reverse the enhanced efferocytosis, reduced pro-inflammatory cytokine expression, and neutrophil recruitment into the lung following apoptotic cell instillation in lung fibrosis [33]. Huang et al. [55] used combined administration of AS1517499 and GW9662 to show that the pSTAT6/PPARγ signaling pathway is associated with reducing pro-inflammatory cytokines and enhancing *IL-10* expression at the mRNA level in macrophages. Therefore, the enhanced STAT6 activity is required for recovering PPARγ activation, which normalizes the efferocytic ability of peritoneal macrophages that subsequently leads to the production of pro-resolving mediators that prevent prolonged production of pro-inflammatory mediators and ultimately resolves acute sterile inflammation. Notably, other studies have established PPARγ as an inhibitor of activator protein-1, specific protein 1, and NF-kB-driven pro-inflammatory cytokine transcription [54,55]. Thus, the effect of PPARγ activation may also be mediated via its transrepression of pro-inflammatory mediator transcription [56,57,58].

## 5. Conclusions

In summary, our findings suggest that modulating STAT6 phosphorylation using a pharmacological inhibitor delays the resolution of acute inflammation by suppressing PPARγ expression and activation. Our in vitro data suggest the possibility that AnxA1 is involved in triggering activation of the STAT6/PPARγ signaling pathway in macrophages. Based on in vivo findings with the STAT6-specific inhibitor AS1517499, we hypothesize that STAT6 activation mediates PPARγ expression and activation in peritoneal macrophages to promote efferocytosis and prevent a prolonged inflammatory response. Our results indicate that specifically targeting the STAT6/PPARγ signaling pathway might be a novel therapeutic approach for treating sterile inflammatory diseases, including peritonitis.

## Data Availability

All data are available within the manuscript and upon request to the corresponding author.
